# Functional constipation in Chinese infants: disruptions in gut microbiota and urinary metabolome revealed by a cross-sectional analysis

**DOI:** 10.3389/fmicb.2025.1649995

**Published:** 2025-09-03

**Authors:** Haihong Zhao, Xiuxia Lu, Hua Lou, Yanbin Ye, Hui Ye, Weiwei Li, Ziyu Long, Xuezhi Zhang, Jieling Wu, Ji Wang

**Affiliations:** ^1^Department of Traditional Chinese Medicine and Integrative Medicine, Peking University First Hospital, Peking University, Beijing, China; ^2^Department of Children’s Health Care, Guangdong Women and Children Hospital, Guangzhou, China; ^3^Department of Hepatobiliary Spleen and Gastroenterology, Zhengzhou Hospital of Traditional Chinese Medicine, Henan University of Chinese Medicine, Zhengzhou, China; ^4^Faculty of Chinese Medicine Science, Guangxi University of Chinese Medicine, Nanning, China; ^5^National Institute of Traditional Chinese Medicine Constitution and Preventive Treatment of Disease, Beijing University of Chinese Medicine, Beijing, China

**Keywords:** age, functional constipation, gut microbiota, infant, urinary metabolome

## Abstract

**Background:**

Functional constipation (FC) is regarded as the most prevalent functional gastrointestinal disorder among children and is closely related to the intestinal microbiota. Nevertheless, there is a paucity of data regarding this relationship in Chinese infants.

**Methods:**

In this study, we investigated the alterations in gut microbiota of 79 FC infants under 2 years of age in China, using 31 balanced non-constipated (BC) children as controls. Some clinical parameters were evaluated, fecal microbiota was analyzed using 16S rRNA gene sequencing, and urinary metabolites were profiled via UPLC-Q-TOF/MS method.

**Results:**

Children with FC demonstrated significantly lower stool scale scores compared to BC group. Significant differences were observed in both microbial community composition and metabolite profiles between the FC and BC groups. The characteristic microbiota of the FC group included *Prevotella*, *Alistipes*, *Collinsella* and *Eggerthella*, associated with dietary fiber degradation, short-chain fatty acid and bile acid conversion, and participated in sphingolipid, thiamine, and glutathione metabolism. A combined biomarker exhibited excellent capacity in distinguishing FC from BC.

**Conclusion:**

This study offers new insights into the gut microbiota and metabolomic profiles associated with FC in young children, particularly that glutathione metabolism may be a new pathway for infant constipation, underscoring the potential for developing innovative therapeutic strategies through the integration of multi-omics approaches.

## 1 Introduction

Functional constipation (FC) represents a prevalent bowel disorder worldwide, affecting individuals across all age groups. It is defined by recurrent symptoms of infrequent stools, difficulty in stool passage, or a sensation of incomplete evacuation. Beyond its gastrointestinal manifestations, FC has been associated with various systemic conditions, including colorectal cancer and cardiovascular diseases (Wu X. et al., 2024). In pediatric populations, organic causes of constipation are exceedingly rare, with epidemiological studies indicating that over 90% of cases are attributed to functional origins. Globally, the prevalence of FC in children, when diagnosed according to the Rome IV criteria, is estimated at 14.4% (Tran and Sintusek, 2023). This condition stands as the most common gastrointestinal disorder in children, with the majority of cases emerging during infancy and toddlerhood (Chogle et al., 2016; Malowitz et al., 2016; Benninga et al., 2016). The long-term implications of untreated or inadequately managed childhood constipation are significant. It has been reported that approximately 25% of affected individuals may experience persistent symptoms extending into adulthood, leading to substantial impairments in quality of life (Pijpers et al., 2010). Early recognition and appropriate intervention are therefore critical in mitigating both the immediate and long-term burdens of this condition.

The infant gut microbiota plays a pivotal role in shaping health across various developmental stages and exerts long-term influences throughout life (Pantazi et al., 2025; Sarkar et al., 2021). In this study, we conducted a cross-sectional analysis involving children aged 0–2 years, recruiting participants with FC as well as those without FC (referred to as BC for balanced/healthy controls). Fecal and urinary samples were collected from these participants and analyzed. This dual approach was utilized to characterize the gut microbiota profiles in FC children and to elucidate the underlying metabolic mechanisms associated with this condition. The findings may provide critical insights for developing targeted interventions and improving clinical outcomes in pediatric populations affected by FC.

## 2 Materials and methods

### 2.1 Study design and participants information

This study adopted a cross-sectional research design. Children aged 0–2 years from Guangdong and Beijing, China, were recruited through poster posting and social media from November 2020 to April 2023. FC was diagnosed according to the Rome IV criteria, which require the presence of at least two of the following symptoms for a minimum of 1 month: (1) two or fewer defecations per week; (2) a history of excessive stool retention; (3) a history of painful or hard bowel movements; (4) a history of large-diameter stools; (5) the presence of a large fecal mass in the rectum. For toilet-trained children, additional criteria include: (1) at least one episode of fecal incontinence per week after acquiring toileting skills; (2) a history of large-diameter stools that have obstructed the toilet. These standardized criteria ensure accurate identification of FC in pediatric populations (Sarkar et al., 2021). BC children referred to those who did not suffer from constipation and were classified as having a balanced constitution according to the Constitutional Medicine Questionnaires (Wang et al., 2019); FC children were classified as having a constitution other than a balanced one.

**Figure 1:**
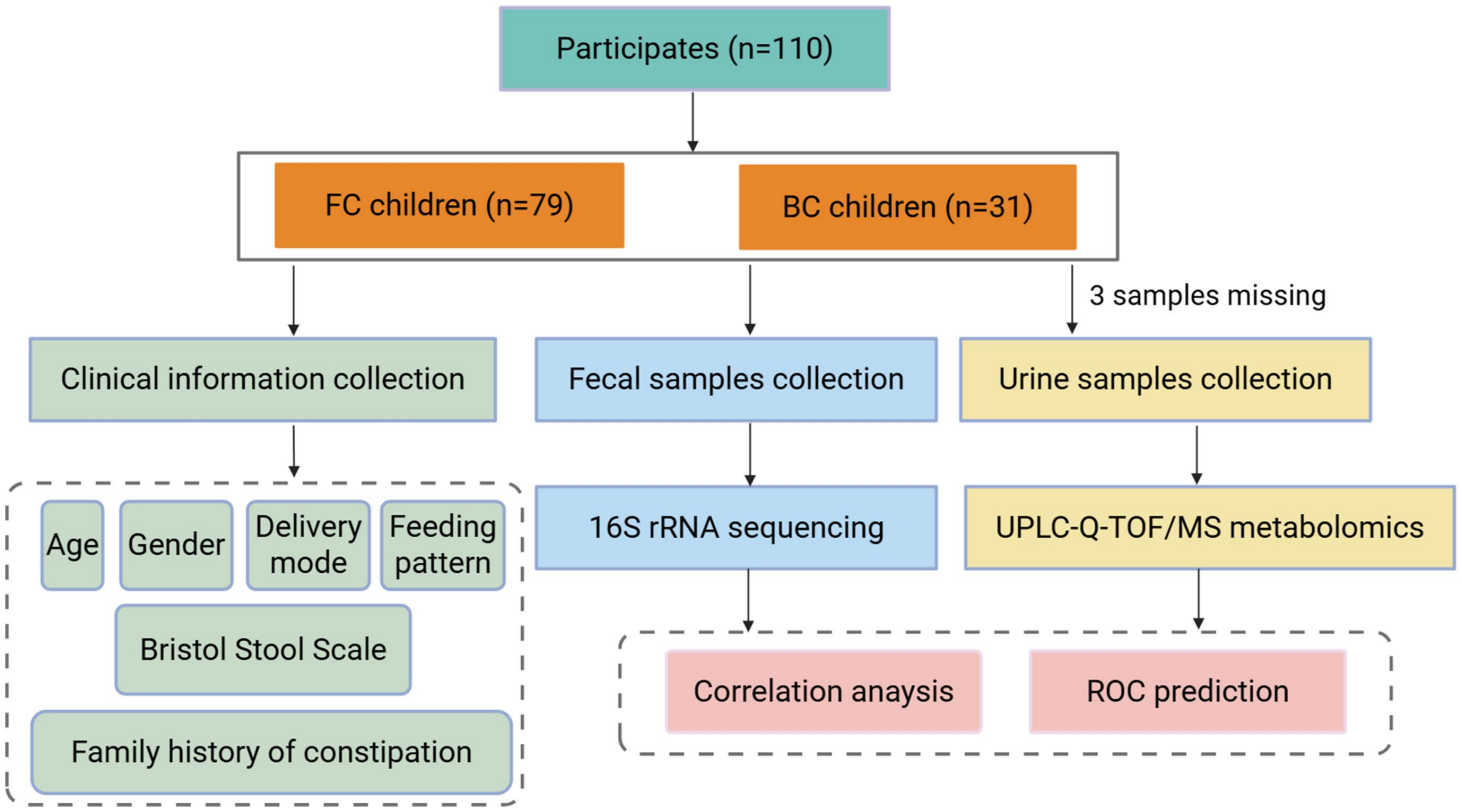
The study flow chart. FC, functional constipation; BC, balanced controls.

Children classified as FC and BC were included in the fecal flora and urinary metabolomics study. Participants who had used antibiotics or probiotics within 3 months prior to sample collection, undergone gastrointestinal surgery, or suffered from neuropsychiatric disorders or other severe conditions were excluded from the study. The study was approved by the Ethics Committee of Beijing University of Chinese Medicine (Approval No. 2020BZYLL122). All guardians of the subjects were provided with detailed information regarding the trial procedures and provided written informed consent. The study flow chart was presented in Figure 1.

### 2.2 Fecal sample collection, DNA extraction and 16S rRNA sequencing

Fecal samples were collected and processed following standardized procedures, as previously described (Lv et al., 2023). Initially, the child’s guardian collected freshly produced, urine-free fecal samples at home and transported them to the hospital within 1 h, maintaining low temperatures using an ice pack. Upon arrival, the samples were immediately flash-frozen in liquid nitrogen and subsequently stored at −80 °C for long-term preservation. Genomic DNA was extracted from the fecal samples using the CTAB/SDS method. The quality and concentration of the extracted DNA were assessed via agarose gel electrophoresis. The hypervariable V3–V4 region of the 16S rRNA gene was amplified through PCR, and the resulting amplicons were purified to construct sequencing libraries. Finally, the libraries were sequenced on the Illumina MiSeq platform to generate high-throughput sequencing data for analysis.

### 2.3 Gut microbiota analysis

Sequencing analysis was performed using the UPARSE software following the merging of paired-end reads and assignment to respective samples. Sequences with similarity ≥97% were grouped into the same operational taxonomic unit (OTU). The α and β diversity of the microbial community were evaluated using QIIME software. To identify differentially abundant microbial taxa, LEfSe analysis was conducted, with a threshold of LDA score > 2.0. Additionally, PICRUSt software was employed to predict functional differences by analyzing gene composition based on the KEGG database.

### 2.4 Urine samples collection and UPLC-Q-TOF/MS processing

The middle portion of morning urine was collected at home by the child’s guardian and transported to the hospital within 1 h, maintained at a low temperature using an ice pack. Upon arrival, the samples were centrifuged, and the supernatant was removed for rapid freezing and stored at −80 °C. For metabolite extraction, the supernatant samples were shipped on dry ice to Shanghai Applied Protein Technology Co., Ltd. for further processing (Wen et al., 2021). Briefly, the samples were thawed, mixed with methanol/acetonitrile solvent, sonicated, and centrifuged. The resulting supernatant was collected and dried, then reconstituted in acetonitrile/water solvent, vortexed, and centrifuged again. Finally, the last supernatant was analyzed using ultra-high-performance liquid chromatography (UHPLC) coupled with quadrupole time-of-flight mass spectrometry (Q-TOF MS) employing a TOF 5600+ system. This comprehensive workflow ensured accurate and reliable metabolomic profiling of the urine samples.

### 2.5 Metabonomics data analysis

For data analysis, the mzXML format was utilized for data processing (Chambers et al., 2021). XCMS was applied for peak alignment, retention time correction, and peak area extraction (Jia et al., 2018). Subsequently, the data underwent metabolite structure identification and preprocessing, followed by quality evaluation of experimental data and final statistical analysis. Univariate analysis, exemplified by fold change analysis, was employed as one of the most commonly used statistical methods. Orthogonal partial least squares discriminant analysis (OPLS-DA) was conducted for multivariate analysis to identify global metabolic differences between the FC and BC groups. The variable importance in projection (VIP) was used to determine characteristic metabolites in the two groups. MetaboAnalyst web software was utilized for metabolic pathway analysis, and Fisher’s exact test was applied to evaluate the significance level of enriched pathways.

### 2.6 Correlation analysis, biomarker identification and statistical analysis

Spearman analysis were utilized to show the correlation between differential flora and metabolites in the two groups by R and Cytoscape software. The area under a receiver operating characteristic (ROC) curve (AUC) was adopted to mine markers of flora and metabolites in FC children. Statistical analyses were performed using SPSS and GraphPad Prism software. *p* < 0.05 was taken as a significant difference.

## 3 Results

### 3.1 Clinical information collection of participants

A total of 79 FC children and 31 BC children were enrolled in the study after data screening and FC diagnosis. It’s well known that age, gender, delivery mode, and feeding pattern can influence the gut bacterial composition (Cryan et al., 2019); in this study, the above variables were statistically analyzed in the FC and BC groups and the results showed no significant differences (Table 1).

**Table 1:** Clinical parameters of the FC and BC groups.

Variables		FC (*n* = 79)	BC (*n* = 31)	*p-*value
Age, mean (SD)	Months	17.32 (4.21)	19.06 (4.88)	0.757
Gender, *n* (%)	Boys	37 (46.84%)	17 (54.84%)	0.450
	Girls	42 (53.16%)	14 (45.16%)	
Delivery mode, *n* (%)	Vaginal delivery	45 (56.96%)	17 (54.84%)	0.714
	Caesarean delivery	34 (43.04%)	14 (45.16%)	
Feeding pattern, *n* (%)	Breast feeding	45 (56.96%)	20 (64.52%)	0.468
	Non-breast feeding	34 (43.04%)	11 (35.48%)	
Family history of constipation, *n* (%)	Yes	21 (26.58%)	1 (3.23%)	<0.001
	No	58 (73.42%)	30 (96.77%)	
Bristol Stool Scale score, *n* (%)	Separate hard lumps, nuts-like	15 (18.99%)	6 (19.35%)	
	Sausage-shaped but lumpy	32 (40.51%)	0 (0.00%)	
	Sausage-shaped with cracks	22 (27.85%)	3 (9.68%)	
	Smooth snake	7 (8.86%)	6 (19.35%)	
	Soft blobs	2 (2.53%)	12 (38.71%)	
	Fluffy pieces	1 (1.27%)	4 (12.90%)	
	Watery	0 (0.00%)	0 (0.00%)	
	Total score, mean (SD)	2.39 (1.06)	3.97 (1.68)	0.002

In addition, Bristol Stool Scale (BSS), were used to evaluate stool consistency. For BC group, the predominant stool shapes were soft blobs, followed by smooth snake and snake-like forms. These characteristics may be attributed to the young age and the typically thinner diameter of feces in this population. In contrast, FC children predominantly exhibited sausage-shaped stools with cracks, a few with fluffy pieces and watery feces. This pattern was influenced not only by age-related characteristics but also by FC. Compared to BC children, the BSS scores were significantly higher in the FC group (*p* = 0.002). Considering whether constipation is genetically influenced, we conducted a family history survey, and the results showed that FC children were more likely to have a family history of constipation than the ordinary children did (*p* < 0.001).

### 3.2 Changed microbial community diversity of FC children

**Figure 2:**
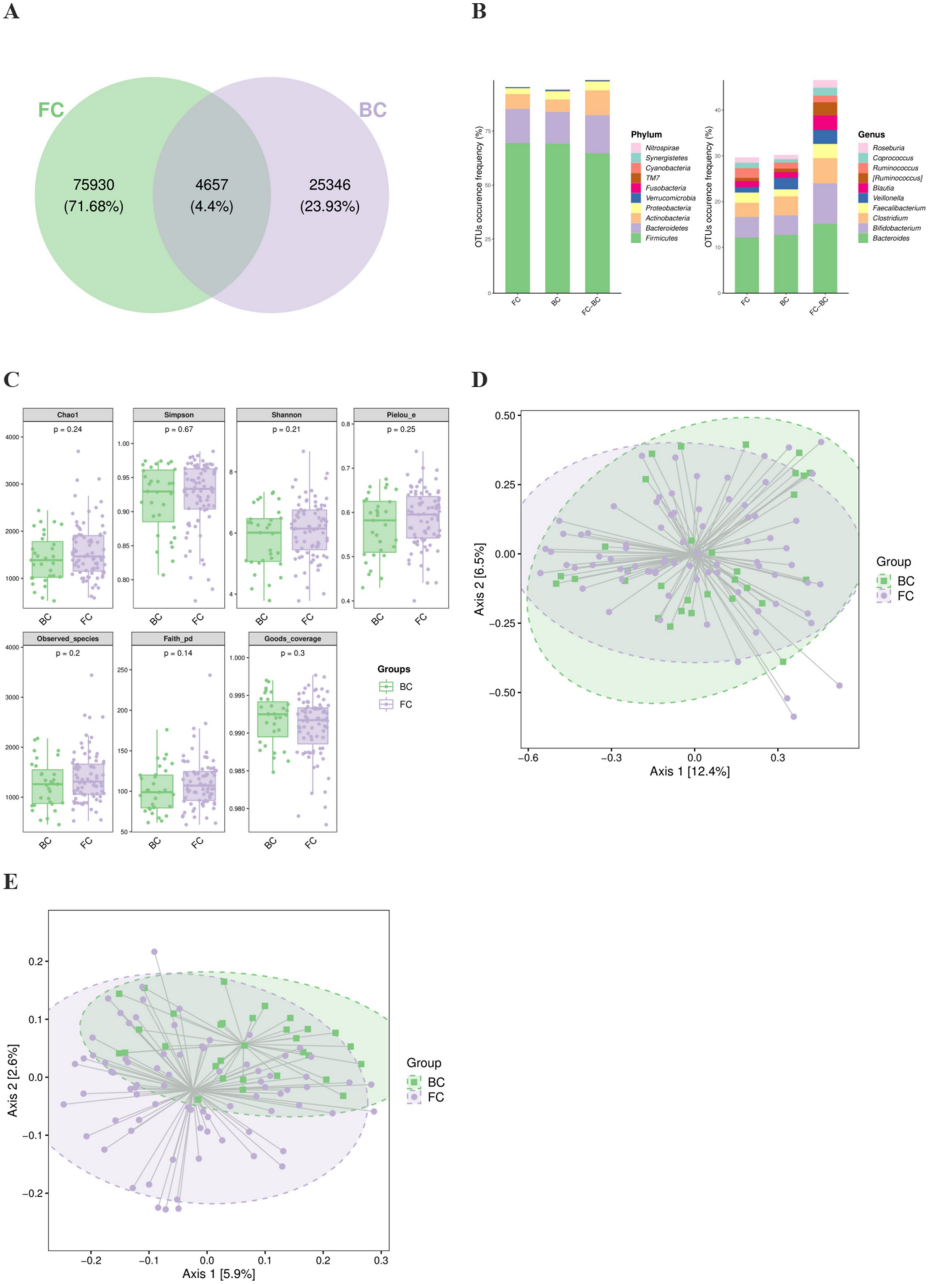
Gut microbial diversity of FC and BC children. **(A)** Venn diagram of ASVs in the FC and BC groups. **(B)** OTU occurrence frequency in two groups. **(C)** α diversity consisting of many indicators. **(D)** β diversity based on weighted UniFrac. **(E)** β diversity based on unweighted UniFrac.

Fecal samples from the FC and BC groups were processed using the DADA2 method. Each sequence generated after DADA2 quality control is referred to as an amplicon sequence variant (ASV), representing a precise microbial sequence. This approach is more accurate than the traditional 97% similarity-based OTU clustering (Callahan et al., 2016). The FC and BC groups contained 75,930 and 25,346 specific ASVs, with 4,657 overlapping ASVs (Figure 2A). OTU occurrence frequency were shown in phylum and genus levels (Figure 2B).

To comprehensively evaluate bacterial diversity within the samples, α diversity analysis was conducted. No statistically significant differences were observed between the FC and BC groups for any of α diversity metrics (*p* > 0.05). This indicates that there was no substantial difference in the internal diversity of the bacterial communities between the two groups (Figure 2C).

To investigate the differences in microbial community composition between groups, β diversity analysis was conducted. Principal coordinate analysis (PCoA), based on weighted and unweighted UniFrac distance as representative metrics, was used to examine the clustering patterns of microbial communities in the FC and BC groups. The results demonstrated a distinct separation between the microbial profiles of the FC and BC groups (PERMANOVA *p* = 0.021, *p* = 0.001), suggesting significant differences in bacterial composition between the two groups (Figures 2D,E).

### 3.3 Discriminative taxa between the FC and BC groups

**Figure 3:**
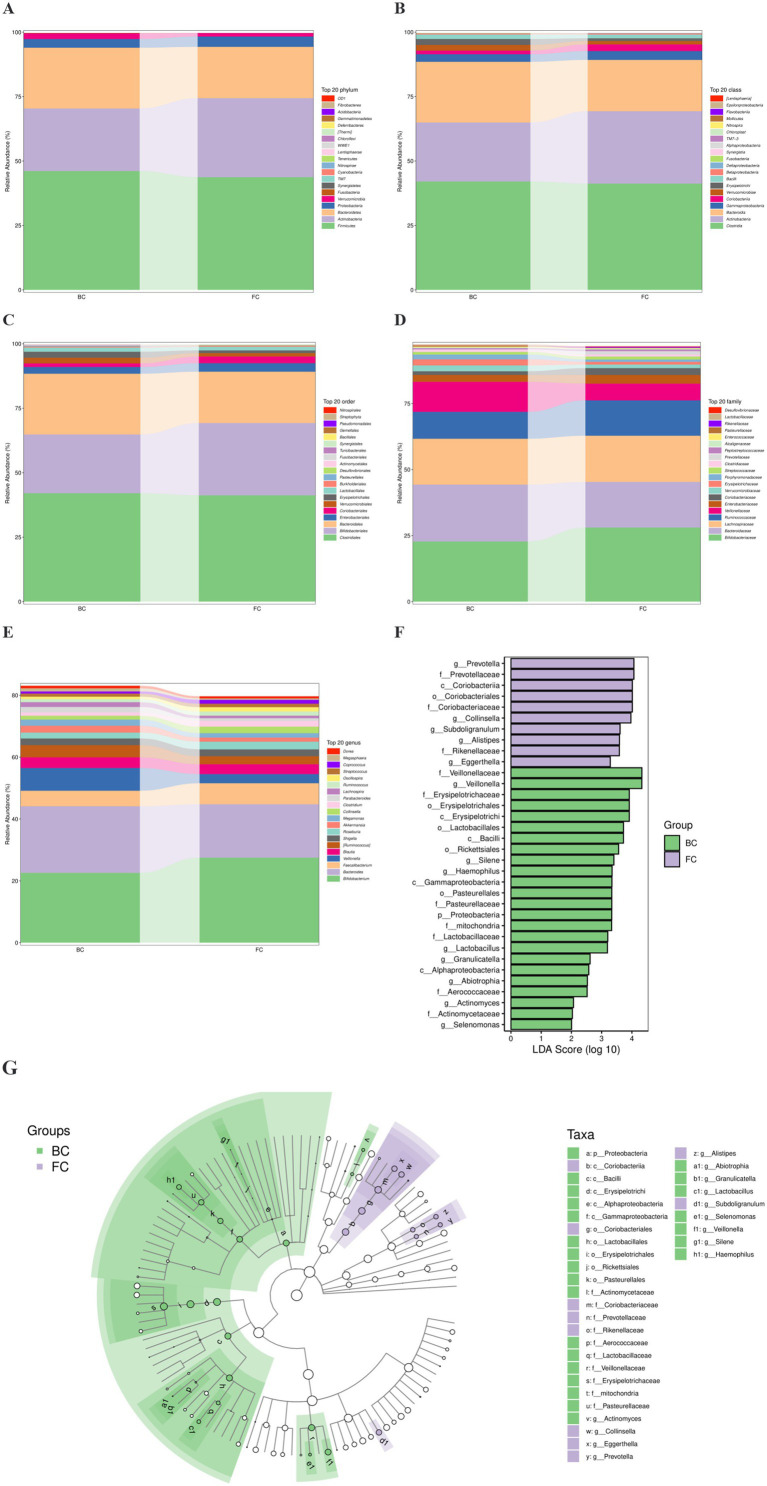
FC children changed the gut microbial composition. **(A–E)** Histogram showing the relative abundance of the top 20 taxa in phyla, class, orders, family, genera, respectively. **(F)** LEfSe analysis showing gut flora with an LDA (log10) >2. The length of the bar graph representing the size of the LDA. **(G)** Cladogram of the phylogenetic distribution at all taxonomic levels.

The gut flora composition was analyzed at five taxonomic levels, namely, phylum, class, order, family, and genus, and the top 20 flora were listed at each level (Figures 3A–E). LEfSe analyses allowed simultaneous analysis of differences at all taxonomic levels to be presented via the cladogram (Figure 3G), and placed considerable emphasis on finding robust biomarkers across groups (Figure 3F). A total of 34 characteristic flora were found, of which 10 were detected in the FC group and 24 in the BC group. In the FC group, the characteristic flora included class Coriobacteriia, order Coriobacteriales, family Coriobacteriaceae, and at the genus level were *Prevotella*, *Collinsella*, *Subdoligranulum*, *Alistipes* and *Eggerthella*. In the BC group, the characteristic flora consisted of class Erysipelotrichi, order Erysipelotrichales, family Erysipelotrichaceae, Lactobacillaceae and Veillonellaceae, and the characteristic genera included *Veillonella*, *Silene*, *Haemophilus*, *Lactobacillus*, *Granulicatella*, *Abiotrophia*, *Actinomyces*, *Actinomycetaceae* and *Selenomonas*.

### 3.4 Altered metabolomics profile of FC children

Most intestinal bacteria exert an influence on the physiological and pathological state of the organism through their metabolites. Urine, as the end product of human metabolism, contains a rich array of metabolites that reflect the body’s biochemical metabolic status. It is also non-invasive, non-infectious, and highly suitable for analyzing metabolic changes in young children. In this study, urine samples were collected from 31 BC children and 76 FC children for untargeted metabolomic analysis.

**Figure 4:**
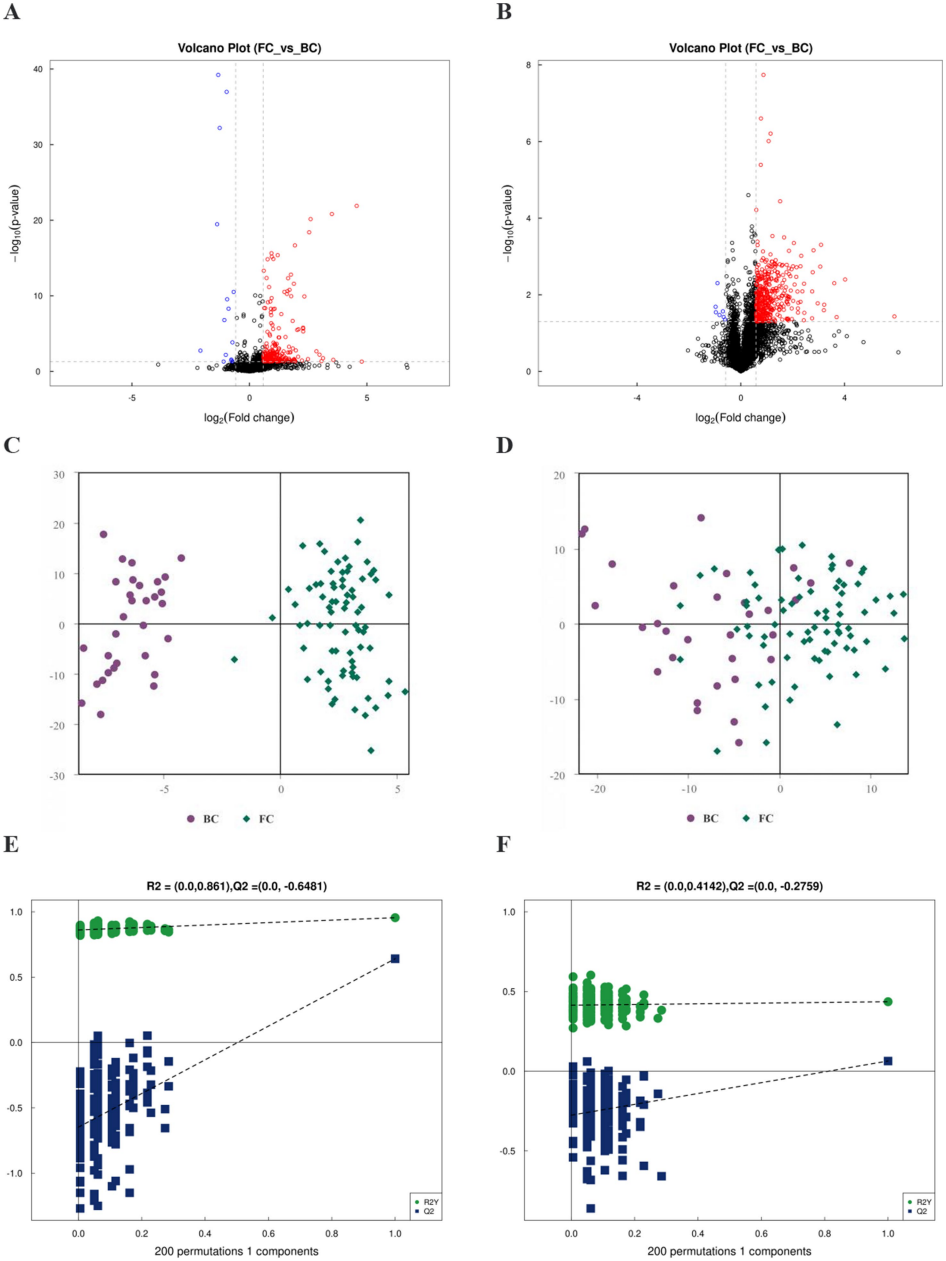
FC children changed the metabolome. **(A)** Volcano plots of positive ion mode. **(B)** Volcano plots of negative ion mode. Rose represented differential metabolites with Fold change >1.5, suggesting up-regulation of their expression in the FC group. Blue represented differential metabolites with Fold change <0.67, suggesting down-regulation of their expression in the BC group. Black represented metabolites with no statistically significant difference. **(C)** OPLS-DA score graph for positive ion mode. **(D)** OPLS-DA score graph for negative ion mode. **(E)** Permutation test of OPLS-DA for positive ion mode. **(F)** Permutation test of OPLS-DA for negative ion mode.

After removing the ineligible samples, the total ion flow plots of the quality control (QC) samples showed substantial overlap, indicating minimal machine-induced errors throughout the experiment (Supplementary Figure S1). Differential analysis of all metabolites was performed using univariate statistical analysis and visualized through volcano plots (Figures 4A,B). OPLS-DA revealed an overall trend of separation between FC and BC samples (Figures 4C,D), suggesting that the OPLS-DA model effectively differentiated the two groups. The OPLS-DA permutation test in the positive and negative ion modes indicated that the original model did not exhibit overfitting and had excellent explanatory and predictive capabilities (Figures 4E,F). These findings indicate that the metabolite composition in FC children underwent significant alterations compared to BC children.

### 3.5 Differential metabolite and metabolic pathway between two groups

**Figure 5:**
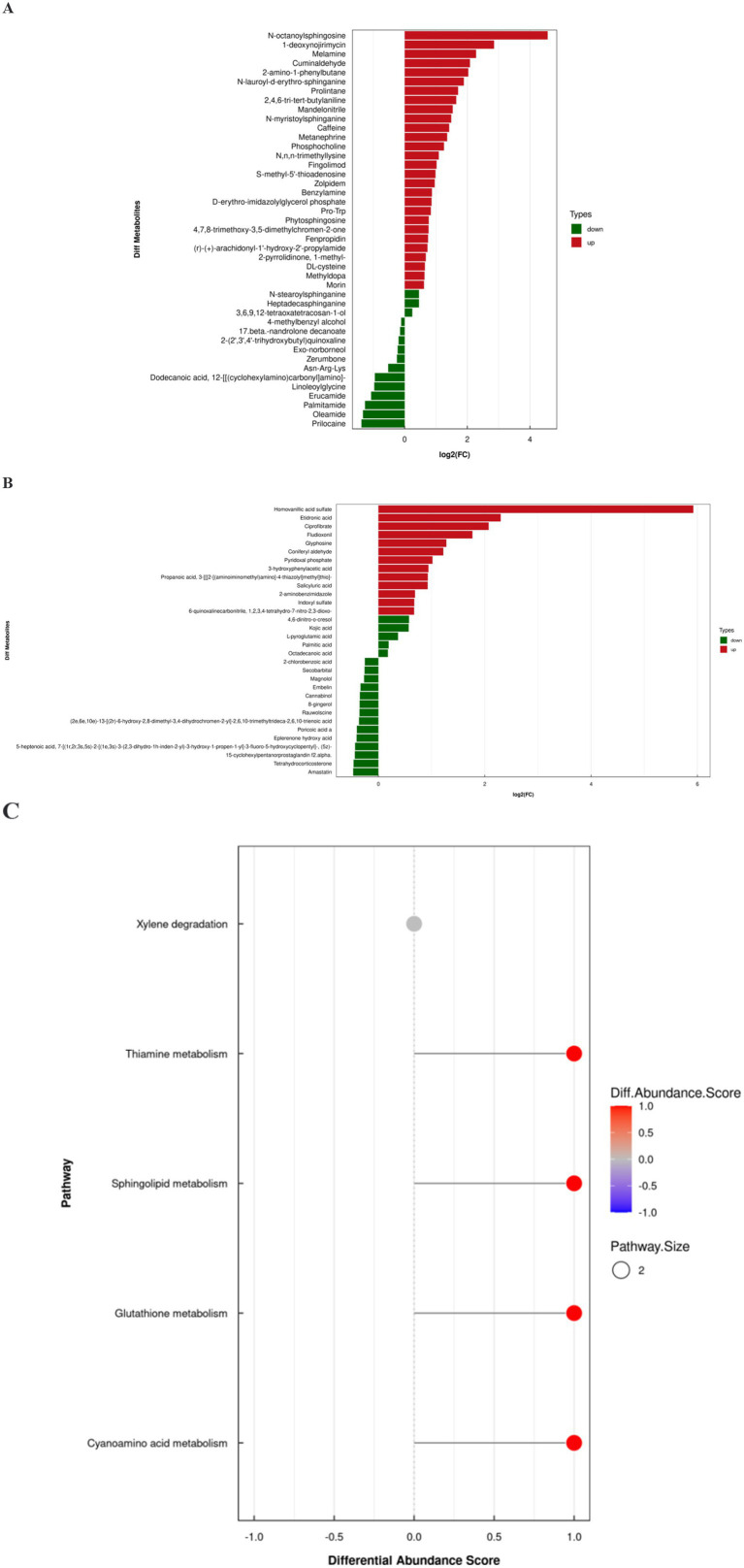
Differential metabolites analysis. **(A)** Differential metabolites in positive ion mode. **(B)** Differential metabolites in negative ion mode. **(C)** Differential abundance score plot of differential metabolites.

The differences in metabolite expression profiles between groups can be quantified and analyzed using variable importance in projection (VIP) values derived from OPLS-DA, allowing for the identification of significantly altered metabolites. In this study, a stringent screening criterion of VIP >1.0 and *p* < 0.05 was applied to select differential metabolites. As illustrated in Figures 5A,B, 43 metabolites were identified in positive ion mode and 32 in negative ion mode. Detailed parameters of each differential metabolite are provided in Supplementary Table S1.

To further explore the metabolic mechanisms of FC, the differential metabolites were mapped to relevant physiological pathways. Differential abundance scores indicated that the FC group was mainly associated with alterations in sphingolipid metabolism, thiamine metabolism and glutathione metabolism (Figure 5C). These findings provide insights into the potential metabolic alterations and biological processes involved in FC.

### 3.6 Correlation analysis between differential flora and metabolites

**Figure 6:**
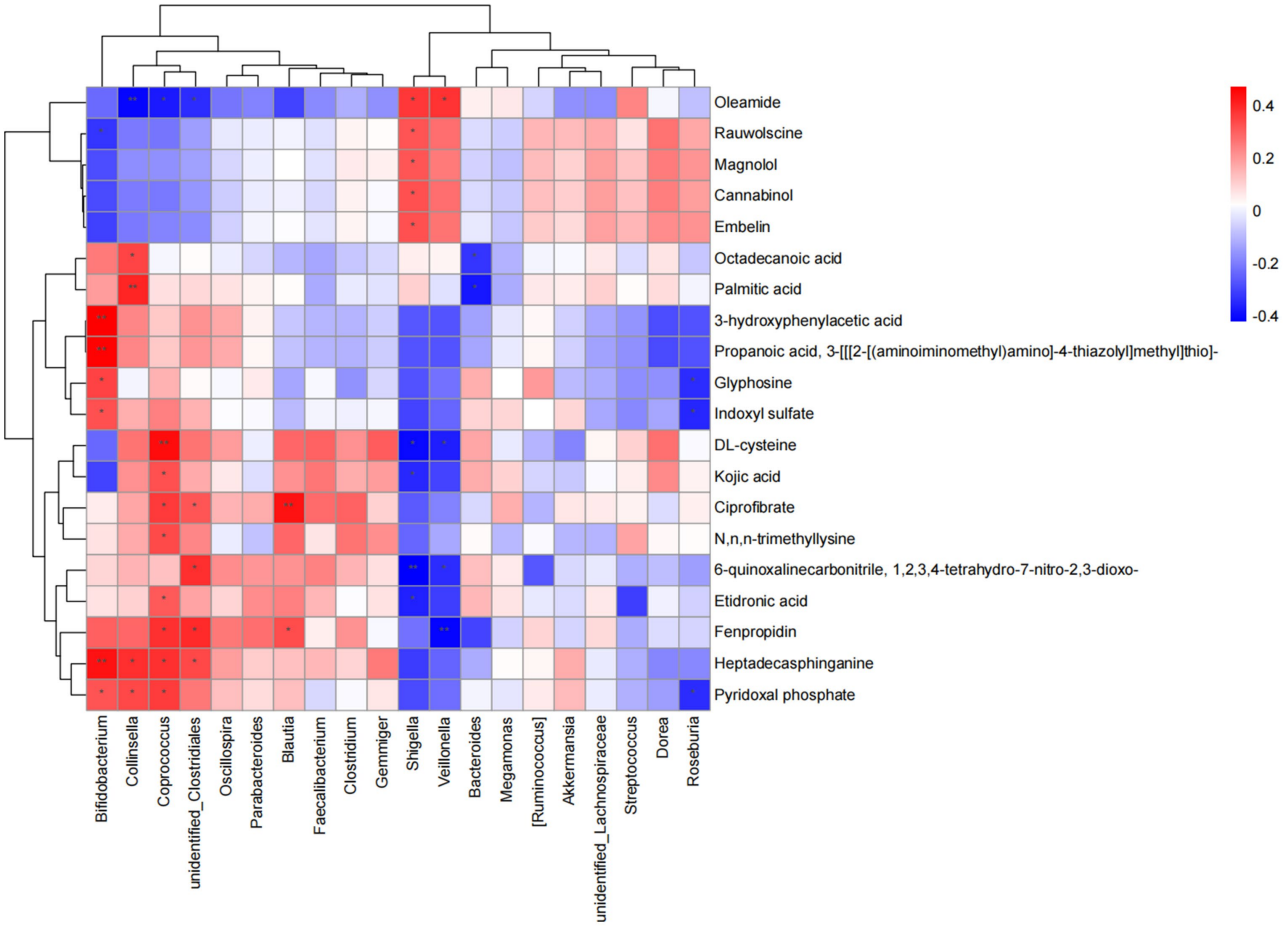
Correlation analysis between differential flora and metabolites. ^*^p < 0.05, ^**^p < 0.01, and ^***^p < 0.001.

To investigate the correlation between gut microbiota and metabolic changes, Spearman analysis was performed. A total of 223 significant pairs were identified. Specifically, These colonies (*Veillonella*, *Collinsella*, *Bifidobacterium*, *Coprococcus*, *Shigella*) and metabolites (oleamide, ciprofibrate, fenpropidin, heptadecasphinganine, pyridoxal phosphate) played tightly connected roles (Figure 6). These findings suggest that specific gut microbes may play a role in regulating the levels of certain metabolites, potentially influencing the metabolic profile observed in FC individuals.

### 3.7 ROC prediction for FC children

**Figure 7:**
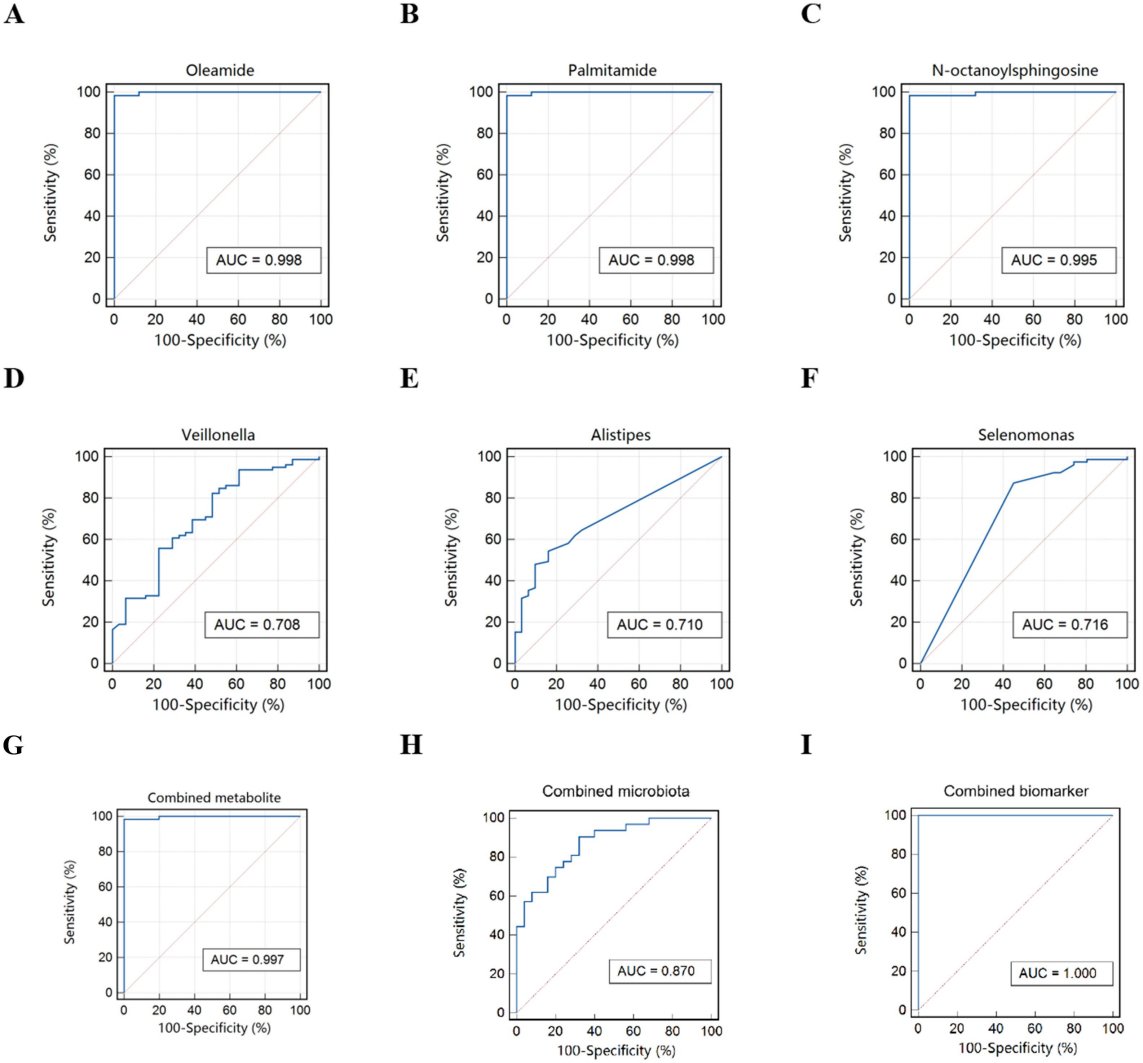
ROC curve for FC children. **(A–C)** Differential metabolites. **(D–F)** Differential microbiota. **(G)** Combined metabolite. **(H)** Combined microbiota. **(I)** Combined biomarker of microbiota and metabolites.

To identify potential biomarkers for FC children, ROC analysis was employed. The top 3 metabolites were Oleamide, Palmitamide, N_octanoylsphingosine, showing excellent discriminatory power. The top 3 microbial taxa were *Alistipes*, *Selenomonas* and *Veillonella*, showing general discriminatory power. When combining these metabolites and microbial taxa, the resulting model exhibited excellent discriminatory power with an AUC of 1.000. Moreover, the sensitivity and specificity of this biomarker panel both reached 100%, suggesting that the model can perfectly distinguish potential FC children without any misclassification (Figure 7).

## 4 Discussion

Within the first 2 years of life, a critical period for human development, trillions of microbes and their associated genes undergo assembly and stabilization within the human body, forming the foundation of the gut microbiome (Robertson et al., 2019). Accumulating evidence underscores dysbiosis of the intestinal flora as a pivotal etiological factor in pediatric FC. Recent metagenomic studies have further illuminated the central role of bacterial composition and metabolic potential in the pathophysiology and progression of FC (de Meij et al., 2016; Mancabelli et al., 2017). Building upon these insights, we undertook an investigation into the intestinal microbiota and urinary metabolomics profiles of Chinese children aged 0–2 years diagnosed with FC. This study was designed to systematically characterize the microbial community structure and its associated metabolic signatures in this specific population, thereby providing a targeted understanding of the microbiome-metabolome interactions underlying pediatric FC during early childhood development.

Although the FC and BC groups did not show significant differences in terms of alpha diversity, LEfSe analysis revealed significant differences in the composition of the gut microbiota between the two groups. Specifically, five genera had significantly higher relative abundance in the FC group than in the BC group; these were *Alistipes*, *Eggerthella*, *Prevotella*, *Collinsella*, and *Subdoligranulum*. Genera with higher relative abundance in the BC group included *Veillonella* and *Selenomonas*. *Alistipes*, a prominent genus within the gut microbiota, exerts dual regulatory effects on intestinal motility through tryptophan metabolism. This genus competitively utilizes tryptophan via the kynurenine pathway, thereby limiting substrate availability for serotonin synthesis through the 5-hydroxytryptophan (5-HTP) pathway (Tyrrell et al., 2011). The resultant serotonin deficiency impairs enteric nervous system signaling, directly contributing to delayed colonic transit-a hallmark of FC. Clinical observations reveal elevated mucosal colonization of *Alistipes* in FC patients, correlating with diminished 5-HTP levels and prolonged intestinal transit times (Mearin et al., 2016; Sugitani et al., 2021). Notably, therapeutic interventions involving *Bifidobacterium* species demonstrate constipation-alleviating effects through a tripartite mechanism: enhancing acetate production, promoting *Lactobacillus* proliferation, and specifically suppressing *Alistipes* overgrowth (Wang L. et al., 2017). This microbial modulation rebalances the tryptophan-serotonin axis while improving luminal SCFA profiles, suggesting *Alistipes* abundance serves as both a biomarker and potential therapeutic target in FC pathophysiology (Natividad et al., 2018; Muralidharan et al., 2021).

The gut microbiota composition in Rett syndrome patients with constipation demonstrates a significant enrichment of *Eggerthella*, a potentially pathogenic genus associated with intestinal barrier dysfunction. This dysbiotic state generates a unique SCFA profile independent of constipation status, suggesting intrinsic microbial metabolic reprogramming linked to *Eggerthella* proliferation (Strati et al., 2016). Mechanistically, *Eggerthella* exhibits proinflammatory properties through induction of intestinal epithelial inflammation and compromised barrier integrity, potentially exacerbating gastrointestinal manifestations including bleeding and bloating (Gardiner et al., 2015). Concurrently, *Prevotella* demonstrates microenvironmental adaptability to inflammatory conditions, with its increased abundance in constipated mice models and human cohorts reflecting a symbiotic relationship with proinflammatory states. This genus may perpetuate intestinal dysmotility through sustained inflammatory signaling (Liu et al., 2019; Li et al., 2024). The microbial ecology analysis reveals functional dichotomy between *Veillonella* and *Selenomonas* in gut homeostasis. While *Veillonella*’s dominance in healthy small intestinal ecosystems aligns with its elevated abundance in control groups, its metabolic reliance on organic acid fermentation may paradoxically exacerbate constipation through gas-mediated intestinal distension. In contrast, *Selenomonas* demonstrates therapeutic potential through glucose fermentation pathways, as evidenced by its post-transplant enrichment in fecal microbiota transplantation studies correlating with improved colonic motility and mitigation of constipation-related neuropsychiatric comorbidities (van den Bogert et al., 2013; Yang et al., 2023). These findings collectively highlight the complex interplay between specific bacterial taxa (*Eggerthella*, *Prevotella*) driving inflammatory-metabolic dysfunction and counterregulatory genera (*Selenomonas*) mediating intestinal homeostasis restoration, providing mechanistic insights into microbial contributions to constipation pathophysiology.

The liquid-quantity coupledomics approach utilized in this study revealed a distinct metabolite profile in children with FC. A total of 75 differential metabolites were identified, along with several associated metabolic pathways. Among these, oleamide, a fatty acid amide derived from oleic acid, emerged as a critical regulator of enteric dynamics. Oleamide has been demonstrated to inhibit intestinal motility by activating the cannabinoid receptor CB1 in murine models (Capasso et al., 2005). Traditional Chinese medicine, specifically MaZiRenWan, has been shown to mitigate oleamide-induced reductions in intestinal motility. This effect is likely mediated through enhanced degradation of oleamide via colonic fatty acid amide hydrolase, thereby improving intestinal motility in FC patients (Huang et al., 2020). Other metabolites identified in this study, such as palmitamide and n-octanoylsphingosine, currently lack established correlations with FC, warranting further investigation into their potential roles in the pathophysiology of this condition. These findings underscore the importance of oleamide and its regulatory mechanisms in FC, while highlighting the therapeutic potential of targeted interventions aimed at modulating fatty acid amide metabolism.

Glutathione serves as a critical antioxidant in the body, capable of scavenging free radicals and reducing oxidative reactions, thereby preserving cellular integrity. Additionally, glutathione plays a key role in promoting metabolism, aiding in the elimination of waste and toxins, and enhancing the body’s immune function. Notably, glutathione metabolism also influences intestinal barrier function by modulating oxidative stress and regulating the expression of pro-inflammatory cytokines. In constipated rats, the content of glutathione was decreased. After treatment with traditional Chinese medicine, its content increased, suggesting a correlation with constipation. A novel strain BC99 exerted an anti-constipation effect in adults, which may be related to glutathione metabolism (Wu Y. et al., 2024).

The sphingolipid metabolic axis plays a pivotal role in pediatric constipation pathophysiology, generating bioactive mediators such as sphingosine-1-phosphate (S1P), phytosphingosine, and dihydrosphingosine. These molecules regulate gastrointestinal motility via dual mechanisms: direct modulation of smooth muscle contractility and neuromodulatory effects mediated by interstitial cells of Cajal (ICC) (Maceyka et al., 2012). Notably, S1P exerts mechanistic significance by activating ICC pacemaker activity through calcium-dependent pathways, essential for colonic smooth muscle slow-wave propagation (Wang et al., 2023). Clinically, reduced S1P levels correlate with ICC network dysfunction and impaired propulsive motility, linking sphingolipid dysregulation to constipation (Mostafa et al., 2010). Supporting evidence from aging models demonstrates analogous gastrointestinal dysmotility associated with dihydrosphingosine depletion, while therapeutic restoration of phytosphingosine enhances cholinergic signaling, exerting prokinetic effects. These findings underscore the critical relationship between the sphingolipid metabolic axis and constipation (Choi et al., 2015; Wang S. et al., 2017).

Thiamine, also known as vitamin B1, is widely distributed in most foods. While it is particularly abundant in cereals, meat, fish, shrimp, and yeast, a significant portion of vitamin B1 is lost during food processing. As a result, many processed foods are fortified with thiamine. The absorption of thiamine primarily occurs in the duodenum, where it binds with magnesium ions to form its active state, thiamine pyrophosphate (TPP). TPP serves as an essential cofactor in critical metabolic pathways, such as the citric acid cycle and the pentose phosphate pathway. Low levels of thiamine can impair mitochondrial function, leading to reduced oxidative metabolism and decreased energy production. Research on malnutrition among elderly individuals in nursing facilities has shown that thiamine deficiency is associated with gastrointestinal issues, including nausea, vomiting, and constipation (Volkert et al., 2011). Du et al. (2024) conducted a large-scale survey involving 10,371 participants, and the results demonstrated a significant negative correlation between thiamine intake and the prevalence of constipation, with a more pronounced association observed in males, as well as individuals without hypertension or diabetes. One possible explanation for this phenomenon is that dietary thiamine intake is closely related to stool softening and an increased frequency of colonic peristalsis.

Additionally, the combination of characteristic metabolites and gut flora provided an excellent discriminatory marker for FC children, with an AUC of 1.000. This highlights the potential utility of metabolomic and microbiomic profiles in diagnosing and understanding the underlying mechanisms of functional constipation in this population. These findings underscore the importance of integrating metabolomics and microbiomics in elucidating the pathophysiology of functional constipation and identifying potential therapeutic targets.

This investigation acknowledges three principal limitations requiring methodological refinement in subsequent research. First, while demographic parameters (age, gender, delivery mode, feeding practice) showed intergroup parity, the inherent biological variability of infant cohorts across a broad age spectrum (0–2 years) may introduce developmental confounders. Future studies employing stricter age stratification could enhance phenotypic homogeneity. Second, constrained sample size and regional recruitment bias potentially compromise statistical power and generalizability. Nationwide multicenter cohorts incorporating longitudinal designs would improve external validity and enable detection of subtle microbiota-host interactions. Third, the inherent taxonomic limitations of 16S rRNA sequencing preclude precise species-level microbial characterization, particularly critical for functionally divergent subspecies within constipogenic genera. Implementation of shotgun metagenomics coupled with metabolomic profiling could resolve this taxonomic ambiguity while elucidating microbial metabolic networks. Complementary mechanistic validation through fecal microbiota transplantation models and pharmacological modulation of identified microbial targets would strengthen causal inference. Addressing these limitations will facilitate translation of observational associations into therapeutic strategies for pediatric functional constipation.

## 5 Conclusion

This study establishes an integrated multi-omics framework for characterizing Chinese FC in pediatric populations. Through systematic correlation of gut microbial dynamics with metabolomic perturbations, we identified FC-specific microbial clusters and bioactive metabolites converging on thiamine, glutathione and sphingolipid metabolic dysregulation. The developed microbial-metabolite diagnostic panel demonstrates superior discriminative capacity for FC detection, outperforming conventional symptom-based assessments. This biomarker platform enables non-invasive stratification of constipation through machine learning-driven pattern recognition of multi-omics signatures.

Our findings establish a functional axis connecting microbial sphingolipid metabolism with intestinal neuromuscular regulation, offering three translational applications: (1) the relationship between crucial gut microbiota and oleamide, (2) strategies for metabolic reprogramming of glutathione, and (3) the correlation between mechanism-based metabolites oleamide and glutathione metabolism. This paradigm advances pediatric gastroenterology practice towards microbiome-informed personalized interventions.

## Data Availability

The datasets generated and/or analyzed during the current study are available in the National Center for Biotechnology Information (NCBI) repository. The Sequence Read Archive (SRA) ID is PRJNA1262707 and PRJNA1267060.
