# Supplementary figures and images for: Functional constipation in Chinese infants: disruptions in gut microbiota and urinary metabolome revealed by a cross-sectional analysis
Source: Front Microbiol. 2025 Sep 3;16:1649995. doi: 10.3389/fmicb.2025.1649995 (PMC12442431; doi:10.3389/fmicb.2025.1649995)

| 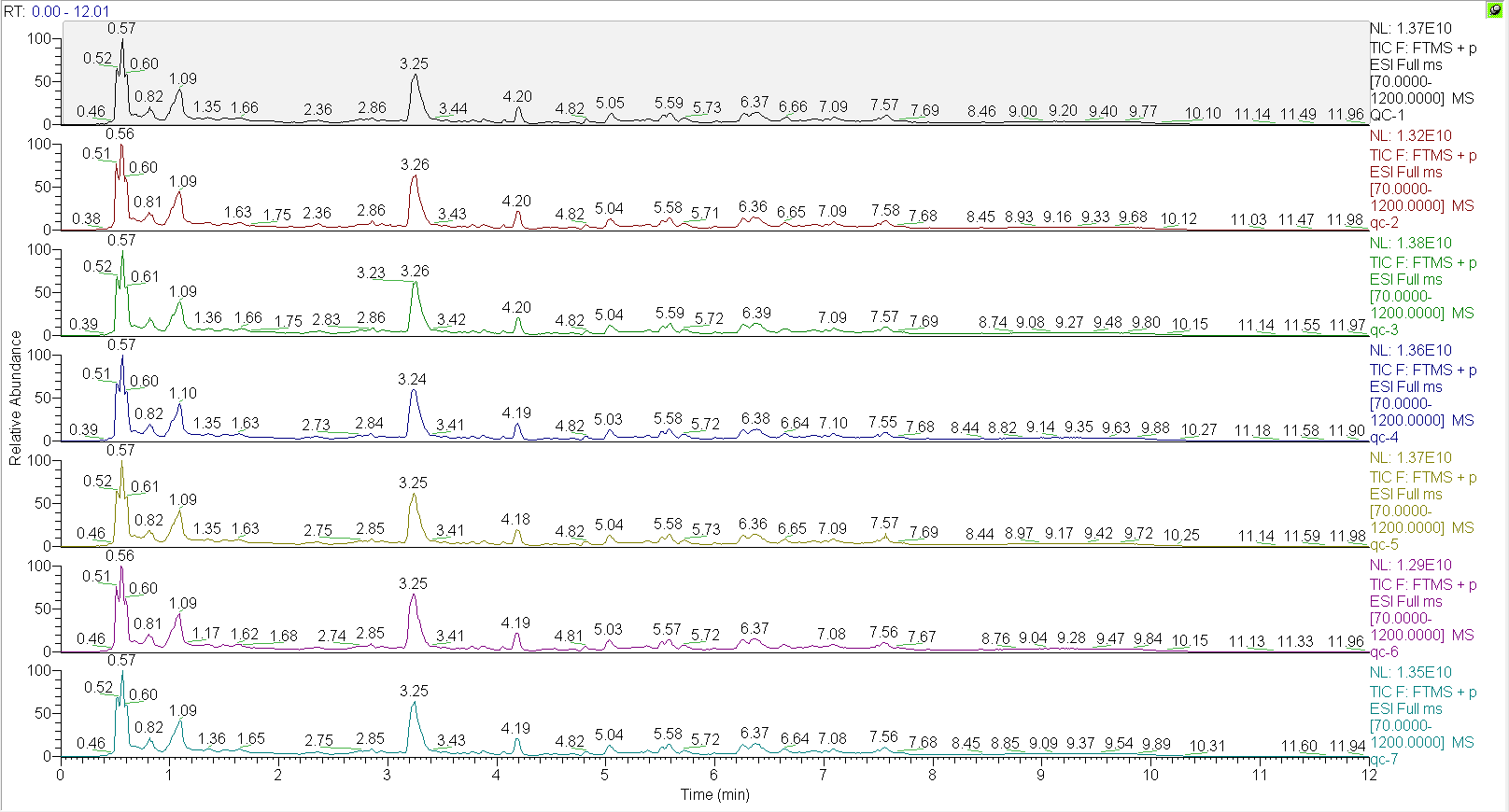 | 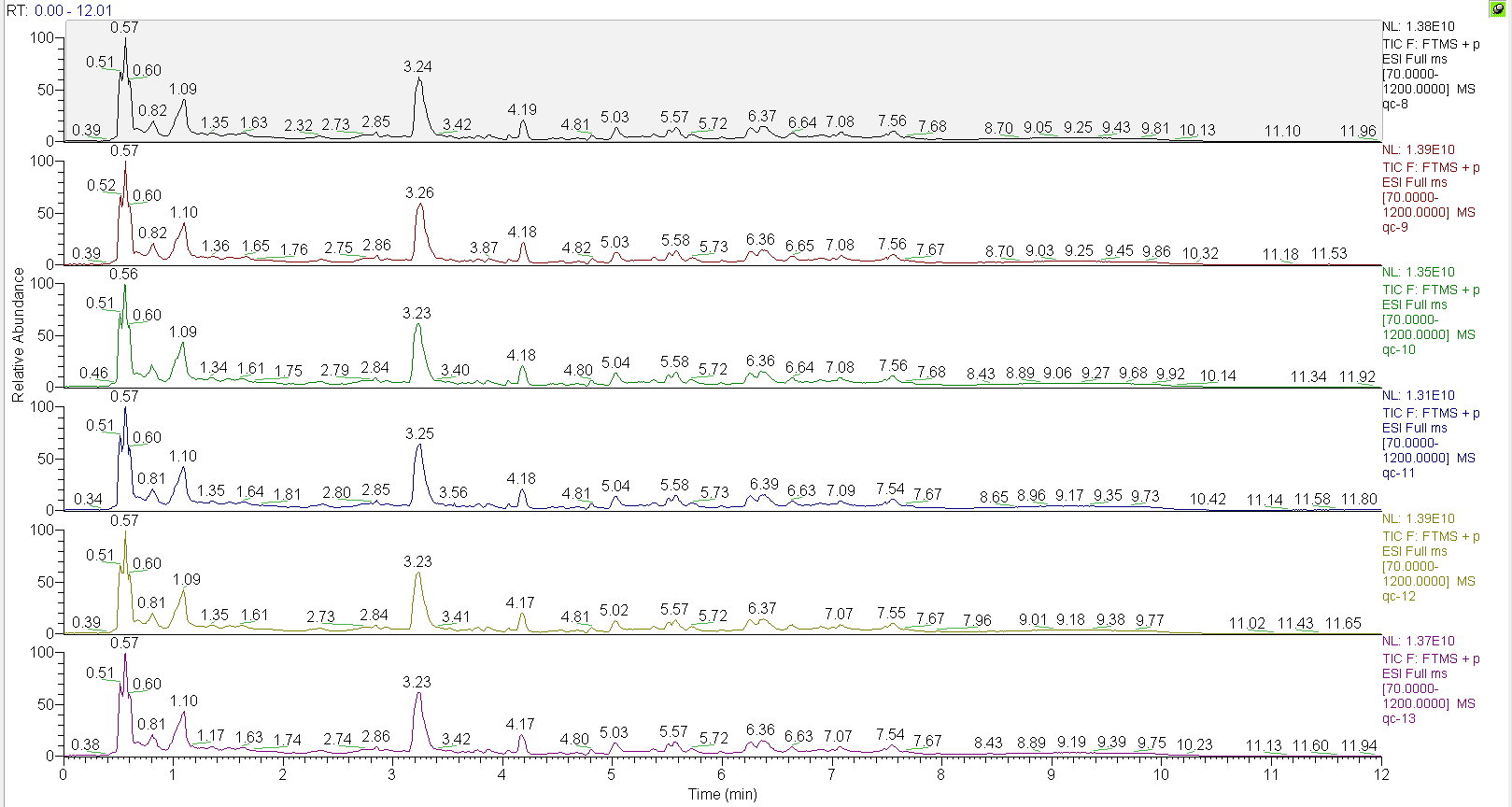 |
| --- | --- |
| **(a)** | **(b)** |
| 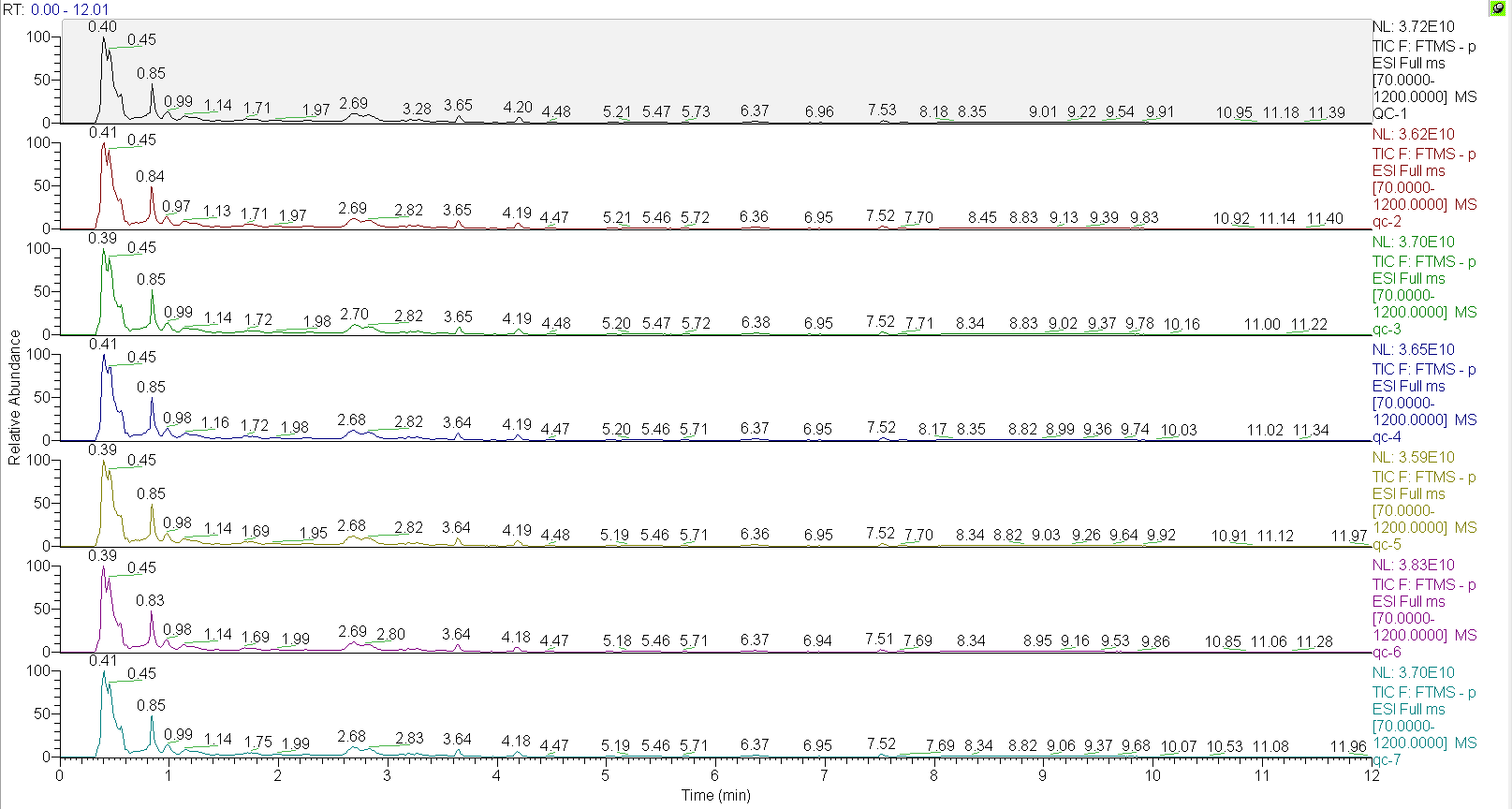 | 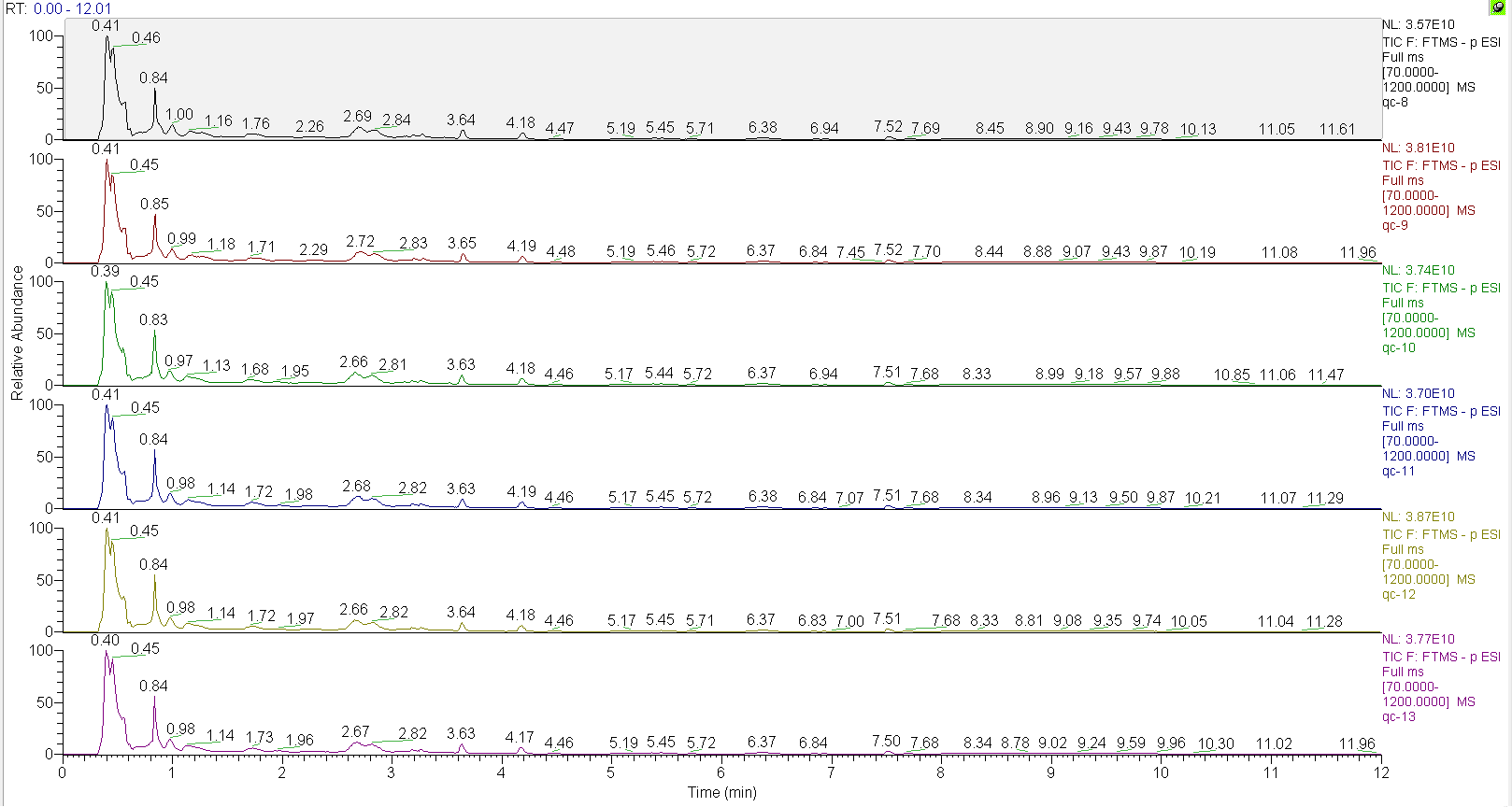 |
| **(c)** | **(d)** |

**Figure S1.** The total ion flow plots of the QC samples. (a,b) positive modes; (c,d) negative modes.

Supplement: Supplementary file 1 [file Supplementary_file_1.docx]
