# Supplementary material for: Functional constipation in Chinese infants: disruptions in gut microbiota and urinary metabolome revealed by a cross-sectional analysis
Source: Front Microbiol. 2025 Sep 3;16:1649995. doi: 10.3389/fmicb.2025.1649995 (PMC12442431; doi:10.3389/fmicb.2025.1649995)
Supplement: Supplementary file 2 [file Supplementary_file_2.docx]

Table S1: Differential metabolites between the FC and BC groups.

| **Name** | **RT(s)** | **m/z** | **Adduction** | **VIP** | ***P-*value** |
| --- | --- | --- | --- | --- | --- |
| (r)-(+)-arachidonyl-1'-hydroxy-2'-propylamide | 68.387900 | 362.32653 | [M+H]+ | 1.216362773 | 4.42352E-13 |
| 1-deoxynojirimycin | 332.634000 | 146.08115 | [M+H-H2O]+ | 2.594620996 | 0.002184616 |
| 17.beta.-nandrolone decanoate | 35.524550 | 429.32055 | [M+H]+ | 1.050145879 | 0.008840088 |
| 2-(2',3',4'-trihydroxybutyl)quinoxaline | 62.371600 | 217.10365 | [M+H-H2O]+ | 1.689816417 | 0.000111426 |
| 2-amino-1-phenylbutane | 33.950100 | 133.10230 | [M+H-NH3]+ | 3.463538873 | 3.26429E-06 |
| 2-pyrrolidinone, 1-methyl- | 57.734100 | 100.07574 | [M+H]+ | 4.478149379 | 0.011241863 |
| 2,4,6-tri-tert-butylaniline | 69.672800 | 246.24277 | [M+H-CH4]+ | 4.77811805 | 4.68915E-13 |
| 3,6,9,12-tetraoxatetracosan-1-ol | 33.098400 | 363.31047 | [M+H]+ | 1.657525211 | 8.88835E-11 |
| 4-methylbenzyl alcohol | 36.754300 | 105.07024 | [M+H-H2O]+ | 2.272117826 | 0.006851968 |
| 4,7,8-trimethoxy-3,5-dimethylchromen-2-one | 250.803500 | 551.21181 | [2M+Na]+ | 1.356714703 | 0.03330276 |
| Asn-Arg-Lys | 33.647000 | 209.15361 | [M+2H]2+ | 1.140035188 | 0.014441241 |
| Benzylamine | 34.565700 | 91.05412 | [M+H-NH3]+ | 2.104531851 | 1.79228E-05 |
| Caffeine | 135.520500 | 195.07640 | [M+H]+ | 1.526701835 | 0.01368122 |
| Cuminaldehyde | 33.925900 | 149.09608 | [M+H]+ | 2.110399674 | 2.67036E-06 |
| D-erythro-imidazolylglycerol phosphate | 70.366900 | 111.05532 | [M+H-CH5O5P]+ | 1.912260047 | 0.007449837 |
| DL-cysteine | 381.757000 | 104.99224 | [M+H-NH3]+ | 2.923444738 | 0.008312213 |
| Dodecanoic acid, 12-[[(cyclohexylamino)carbonyl]amino]- | 33.967300 | 198.18524 | [M+H-C7H13NO2]+ | 1.810490015 | 2.84613E-10 |
| Erucamide | 33.618800 | 338.34176 | [M+H]+ | 4.831570767 | 1.61517E-07 |
| Exo-norborneol | 34.328900 | 95.08554 | [M+H-H2O]+ | 1.307152138 | 0.00039118 |
| Fenpropidin | 64.906600 | 274.27396 | [M+H]+ | 12.34062388 | 0.000533259 |
| Fingolimod | 96.669200 | 290.26896 | [M+H-H2O]+ | 5.103939176 | 1.3361E-15 |
| Heptadecasphinganine | 47.063850 | 288.28971 | [M+H]+ | 3.592791027 | 0.002274231 |
| Linoleoylglycine | 34.558800 | 263.23697 | [M+H-C2H5O2N]+ | 3.802740395 | 1.06425E-37 |
| Mandelonitrile | 63.708600 | 134.08118 | [M+H]+ | 1.058489566 | 2.79772E-05 |
| Melamine | 33.618900 | 127.07535 | [M+H]+ | 1.073411273 | 1.60035E-06 |
| Metanephrine | 107.871500 | 180.08668 | [M+H-H2O]+ | 1.264029326 | 0.016732537 |
| Methyldopa | 327.409500 | 212.10299 | [M+H]+ | 1.353158262 | 0.020747526 |
| Morin | 251.100000 | 303.07281 | [M+H]+ | 1.376978982 | 0.025861647 |
| N-lauroyl-d-erythro-sphinganine | 32.630600 | 484.47234 | [M+H]+ | 2.641975035 | 2.54051E-12 |
| N-myristoylsphinganine | 32.871400 | 512.50363 | [M+H]+ | 5.681750115 | 3.0431E-11 |
| N-octanoylsphingosine | 65.050200 | 390.35757 | [M+H-2H2O]+ | 1.106811169 | 1.26521E-22 |
| N-stearoylsphinganine | 36.740400 | 568.56646 | [M+H]+ | 1.826445618 | 8.78853E-07 |
| N,n,n-trimethyllysine | 577.248000 | 189.15973 | [M+H]+ | 4.568431169 | 0.00036166 |
| Oleamide | 34.064250 | 282.27908 | [M+H]+ | 18.43647508 | 6.05403E-40 |
| Palmitamide | 34.290000 | 256.26348 | [M+H]+ | 5.556719476 | 6.27628E-33 |
| Phosphocholine | 164.221000 | 184.06334 | [M+H]+ | 1.459067865 | 0.046475471 |
| Phytosphingosine | 66.808500 | 318.30021 | [M+H]+ | 3.435800974 | 1.45563E-15 |
| Prilocaine | 52.612200 | 221.16491 | [M+H]+ | 1.126236462 | 3.38789E-20 |
| Pro-Trp | 62.294200 | 302.30532 | [M+H]+ | 4.876297188 | 6.91975E-12 |
| Prolintane | 79.514800 | 218.21139 | [M+H]+ | 4.282478413 | 1.6051E-11 |
| S-methyl-5'-thioadenosine | 98.736500 | 298.09681 | [M+H]+ | 3.564078268 | 7.10336E-07 |
| Zerumbone | 35.976300 | 119.08648 | [M+H-C6H12O]+ | 2.688776167 | 3.00547E-08 |
| Zolpidem | 158.203500 | 235.11898 | [M+H-C3H7ON]+ | 2.527332229 | 0.027353388 |
| (r)-(+)-arachidonyl-1'-hydroxy-2'-propylamide | 68.387900 | 362.32653 | [M+H]+ | 1.216362773 | 4.42352E-13 |
| 1-deoxynojirimycin | 332.634000 | 146.08115 | [M+H-H2O]+ | 2.594620996 | 0.002184616 |
| (2e,6e,10e)-13-[(2r)-6-hydroxy-2,8-dimethyl-3,4-dihydrochromen-2-yl]-2,6,10-trimethyltrideca-2,6,10-trienoic acid | 26.282100 | 425.25741 | [M-H]- | 3.626098077 | 0.033230903 |
| 15-cyclohexylpentanorprostaglandin f2.alpha. | 24.675000 | 365.23627 | [M-H]- | 1.784317023 | 0.013750756 |
| 2-aminobenzimidazole | 27.950800 | 132.04547 | [M-H]- | 1.531432099 | 0.023675421 |
| 2-chlorobenzoic acid | 103.066000 | 154.99056 | [M-H]- | 2.169936674 | 0.022880744 |
| 3-hydroxyphenylacetic acid | 24.442900 | 107.04956 | [M-H-CO2]- | 8.22818172 | 0.002473758 |
| 4,6-dinitro-o-cresol | 135.425000 | 180.03368 | [M-H-HO]- | 1.214090522 | 0.045095511 |
| 5-heptenoic acid, 7-[(1r,2r,3s,5s)-2-[(1e,3s)-3-(2,3-dihydro-1h-inden-2-yl)-3-hydroxy-1-propen-1-yl]-3-fluoro-5-hydroxycyclopentyl]-, (5z)- | 25.419700 | 381.23112 | [M-H-HF]- | 4.09044881 | 0.013280939 |
| 6-quinoxalinecarbonitrile, 1,2,3,4-tetrahydro-7-nitro-2,3-dioxo- | 23.792900 | 203.00192 | [M-H-CO]- | 3.456130636 | 0.04498333 |
| 8-gingerol | 24.478250 | 321.21044 | [M-H]- | 2.793637654 | 0.02583695 |
| Amastatin | 25.887250 | 473.28212 | [M-H]- | 1.708175033 | 0.020491909 |
| Cannabinol | 25.125300 | 309.17404 | [M-H]- | 8.36914761 | 0.020619189 |
| Ciprofibrate | 23.651500 | 287.02299 | [M-H]- | 1.703473633 | 0.005407338 |
| Coniferyl aldehyde | 51.499300 | 162.01965 | [M-H-CH3]- | 1.854108368 | 0.000293214 |
| Embelin | 24.670400 | 293.17914 | [M-H]- | 6.319709196 | 0.02040469 |
| Eplerenone hydroxy acid | 24.850400 | 337.20543 | [M-H-C2H6O4]- | 4.343918601 | 0.01486881 |
| Etidronic acid | 23.137850 | 204.98121 | [M-H]- | 3.128167617 | 0.001803525 |
| Fludioxonil | 31.723750 | 247.02821 | [M-H]- | 3.082627059 | 0.04203749 |
| Glyphosine | 58.861400 | 243.99212 | [M-H-H2O]- | 5.63383699 | 0.042176057 |
| Homovanillic acid sulfate | 220.404000 | 261.00755 | [M-H]- | 1.720643702 | 0.036732611 |
| Indoxyl sulfate | 27.928800 | 212.00225 | [M-H]- | 22.14318586 | 0.011724478 |
| Kojic acid | 382.212000 | 141.01690 | [M-H]- | 1.075251014 | 0.010661542 |
| L-pyroglutamic acid | 327.827500 | 128.03532 | [M-H]- | 1.374442139 | 0.041217898 |
| Magnolol | 25.103800 | 265.14783 | [M-H]- | 12.27951381 | 0.040350481 |
| Octadecanoic acid | 50.434850 | 283.26424 | [M-H]- | 6.316826552 | 0.013197733 |
| Palmitic acid | 50.963600 | 255.23301 | [M-H]- | 5.776813309 | 0.00196781 |
| Poricoic acid a | 26.738900 | 497.31524 | [M-H]- | 1.15023714 | 0.030820722 |
| Propanoic acid, 3-[[[2-[(aminoiminomethyl)amino]-4-thiazolyl]methyl]thio]- | 24.430700 | 187.00775 | [M-H-C3H4O2]- | 41.74183387 | 0.003003783 |
| Pyridoxal phosphate | 34.314900 | 227.99730 | [M-H-H2O]- | 6.520689673 | 0.008405985 |
| Rauwolscine | 25.767800 | 353.19985 | [M-H]- | 7.743896548 | 0.0219105 |
| Salicyluric acid | 99.630000 | 194.04934 | [M-H]- | 1.361807761 | 0.015228269 |
| Secobarbital | 25.539250 | 237.11647 | [M-H]- | 1.162866427 | 0.045129409 |
| Tetrahydrocorticosterone | 24.200100 | 349.24136 | [M-H]- | 1.156532093 | 0.005584776 |
